# Climate change, migration, and health: perspectives from Latin America and the Caribbean

**DOI:** 10.1016/j.lana.2024.100926

**Published:** 2024-10-23

**Authors:** Carolina Batista, Michael Knipper, Ana Cristina Sedas, Sofia Virginia Farante, Daniel Wainstock, Diego B. Borjas-Cavero, Karol Rojas Araya, Juan Carlos Arteaga España, Marisol Yglesias-González

**Affiliations:** a Lancet Migration Latin America Regional Hub, Brazil; b Drugs for Neglected Diseases Initiative, America Latina (DNDi-Latin America), Rio de Janeiro, Brazil; c Institute of the History, Theory and Ethics of Medicine, University Justus Liebig Giessen, Giessen, Germany; d Johns Hopkins University, Bloomberg School of Public Health, Baltimore, MD, United States; e Law School, Pontifical Catholic University of Rio de Janeiro, Rio de Janeiro, RJ, Brazil; f Unidad de Ciudadanía Intercultural y Salud Indígena (UCISI), Facultad de Salud Pública y Administración, Universidad Peruana Cayetano Heredia, San Martín de Porres, 15102, Peru; g Universidad de Costa Rica, San Jose, Costa Rica; h Universitat de Barcelona, Law School, Barcelona, Spain; i Latin American Centre of Excellence for Climate Change and Health, Universidad Peruana Cayetano Heredia, San Martín de Porres, 15102, Peru; j Doctors Without Borders, Latin American Association, Colombia

**Keywords:** Climate change, Migration, Indigenous groups, Global health, Climate justice

## Abstract

This article delves into the complex relationship between climate change, migration patterns, and health outcomes in Latin America and the Caribbean (LAC). While the severe impact of climate change on health in LAC is widely acknowledged, the article sheds light on the often-overlooked multiple effects on migration and the well-being of migrants. These impacts encompass poverty, food and water insecurity, and adverse physical and mental health outcomes. Our paper, guided by a rights-based framework, aims to identify key trends, challenges, and opportunities that can contribute to enhanced knowledge and generate questions to support future research. By emphasizing the need for collaborative efforts across sectors, including public and private entities, civil society, and academic institutions, we aim to address the nuanced intersections of climate change, migration, and health impacts in the region. This approach prioritises the needs of the most vulnerable, including migrants, establishing a framework for mitigation and adaptation that ensures equitable outcomes.

**Search strategy**

References for this Personal View were identified through a literature search using PubMed, Google Scholar, and Scielo.org, with the search terms “climate change”, “migra∗“, “health”, “healthcare”, and “distress migra∗” with and without the regional scope “Latin America” and “Caribbean”. We included Spanish and Portuguese searches to ensure a more inclusive and regionally driven research coverage. The time frame for the publications searched was from January 1, 2000, to March 31, 2024. Papers, monographs, collected volumes and reports e.g. by academic scholars, NGOs and International Organizations published in Spanish, Portuguese and English were considered, and searches of the authors' own files and collections. Haiti was chosen as a case study because it exemplifies the intricate and complex relationships among social, structural, political, and environmental factors affecting decisions to migrate and migrant health outcomes. We also believe in this specific case's potential to shed light on the broader regional challenges ahead.

## Introduction

Migration is more than a movement; it's a complex dance of hope and hardship. The decision to migrate arises from a complex blend of political instability, social inequalities, economic difficulties, and environmental changes. Individual factors such as age, gender, and education, combined with external barriers like immigration laws, further influence each migrant's experience.^1^ Migration can be voluntary, in pursuit of better living conditions and opportunities, or involuntary, driven by external shocks or factors. Regardless of the type, health vulnerabilities can be heightened as migrants confront numerous challenges associated with their journeys. Understanding this complex interplay is key to grasping migration dynamics, particularly in a changing climate.

In this diverse landscape of migration push factors, Latin America and the Caribbean (LAC) grapples with its own set of daunting challenges that drive and shape migration patterns. The region faces a multitude of challenges, encompassing public health, socio-economic issues, and the compounded impacts of climate change and environmental degradation. With more frequent and prolonged droughts, extreme heat waves, increased precipitation, floods, storms, wildfires, and landslides, communities are grappling with the cascading effects of climate change, including extensive losses of homes, infrastructure, crops, livestock, and livelihoods. This has led to millions of people being displaced, while millions more have undertaken the decision to migrate as a response.^2^, ^3^, ^4^, ^5^

While the severe health impacts of climate change and environmental degradation are now widely recognised,^6^, ^7^, ^8^, ^9^, ^10^, ^11^ the intricate relationship between climate change, migration, and health has not yet been considered in sufficient detail. Climate change is not only a major driver of human migration but intersects in multiple ways both with other reasons that make people leave their homes. Regarding health, immediate effects through heat and landslides as well as indirect impacts on the underlying determinants of health affect people before, during, and after the actual migration process, regardless of the reason for migration.^12^ In particular, the LAC region exemplifies this phenomenon, where environmental degradation and the increasing intensity and frequency of slow and sudden-onset weather hazards, compounded with a continuum of push factors, have directly or indirectly impacted people's decision to migrate.^13^ Millions of people are internally displaced due to environmental events, exacerbating humanitarian and developmental challenges in the most vulnerable regions.^9^

In addition, migration in the context of climate change often intersects with other drivers, such as poverty, violence, political instability and the enduring legacy of colonialism that still shapes the region's social fabric.^14^^,^^15^ Embarking on a long and taxing journey as “distress migrants”^16^ exposes them to a myriad of challenges, including deteriorating health conditions due to the direct and indirect effects of climate change. The combination of extreme temperature, limited resources, and restricted access to healthcare along the migration journey, particularly affects the most marginalised, which include children, the elderly, pregnant women, and individuals living with chronic conditions. Coupled with challenges like overcrowded (and inadequate) shelters, food, water shortages, and violent or extreme weather transit experiences, increase marginalised individuals' risk for prolonged illness and even death.

The accumulation of health hazards encountered along migratory journeys during the climate crisis can clearly be seen, for example, in the case of people who left Venezuela. While crossing multiple Andean countries by foot, distress migrants from Venezuela have experienced an increased risk of infectious and vector-borne diseases due to contaminated water during flooding or because of water scarcity during severe drought.^17^ The journey to Central and North America crossing the Darien Gap also provides a stark illustration of how climate change exacerbates the physical and mental health risks faced by migrants in transit. Reports indicate a notable rise in both the number and severity of respiratory and diarrhoea cases.^18^^,^^19^ Additionally, the Darien Gap experienced an increase in precipitation, resulting in elevated water levels, strong river currents, and a heightened risk of landslides. Extreme heat has also made the long trek across the Chihuahuan Desert, through New Mexico and parts of Texas, even more dangerous and deadly. The number of heat-related deaths among migrants crossing this remote desert has been on the rise and between 2022 and 2023, 60 migrants lost their lives due to extreme heat, accounting for just under half the total migrant death toll.^20^ These environmental challenges ultimately amplify the dangers and vulnerabilities faced by migrants, exposing them to severe injuries or even fatalities during transit.

Gender-based violence (GBV) occurs at the various stages of migration, including in countries of origin, transit, and destination. Women, including young girls, face heightened vulnerability to GBV and other forms of abuse during their journey.^21^ Perpetrators of such acts of violence include smugglers, “coyotes”, human traffickers, other migrants and authorities (police, border patrol etc.). GBV experiences are rooted in structural and gender inequalities, and unequal power dynamics, causing devastating effects on victims, their families, and communities. In addition to the physical risk, GBV survivors also experience severe mental health consequences, exacerbating mental distress, anxiety, and fear experienced during migration. Despite the scarcity of literature on LGBTQI migrants in the field of GBV, evidence shows that they face gender-specific forms of violence and discrimination. Furthermore, the lack of safe pathways for GBV survivors to seek protection, worsened by their fear of deportation, imprisonment, and stigmatization, compounds existing risks and vulnerabilities.^22^, ^23^, ^24^

By exploring the interplay between climate change, migration, and health, in this Personal View we delve into the complex dynamics in the LAC region, moving beyond the view of climate change solely as a push factor for migration. The focus is on the broader public health implications of climate change and migration, including the epidemiological shifts, impact on health systems and communities, as well as the physical and mental health effects of climate change, including morbidity, mortality, and psychological implications. Our analysis focuses on the multifaceted relationship between climate change and health determinants among all migrant populations in all phases of the migration process, and regardless of categories defined e.g. through legal provisions or migration policies.

Our perspective is guided, in contrast, by particular attention to people in situations of “distress migration”,^16^ who are forced to leave unbearable living conditions for the sake of a better–and often healthier–future, both within their country (as “internally displaced people”, IDP) and outside (as international migrants). Distress migrants flee desperate conditions due to the intersection of social, economic, political and environmental factors, yet they do not meet the definition of “refugee” according to the 1951 Geneva Convention.^25^ As the climate crisis worsens, the role of environmental and climate factors in driving distress migration will grow, yet the international migration and refugee framework is not yet prepared to provide the protection they deserve. Reflecting the epistemological propositions of critical perspectives in social epidemiology and migrant health,^26^^,^^27^ our approach emphasizes the social, structural, and political determinants of health, migration and environmental degradation. It is grounded in an unwavering commitment to universal human rights, especially the right to the highest attainable standard of health and health equity, as enshrined in various regional and global treaties and political declarations.^28^^,^^29^

## Human mobility and climate change in LAC

Migration flows in LAC are driven by complex and multifaceted factors that are amplified by climate change. The phenomena are deeply intertwined with the region's historical and current socio-political dynamics that are rooted in the legacy of colonialism. The cruel effects of colonialism in LAC include structural racism, forced labour, power privileges, poverty, striking economic inequalities, cultural loss, as well as systematic rights violation and violence against marginalised groups, especially Indigenous and Afro Descendants.^30^, ^31^, ^32^ In recent decades, the region has registered significant increases in human mobility and concentrates 57.5 million migrants, including internally displaced people and asylum seekers, corresponding to approximately 27% of all migrants worldwide.^9^ Drivers of distress migration such as impoverishment, destitution, violence, fragile or weakened institutions with limited health and social protection, are further exacerbated by both acute and slow onset climate-events, leading to limited access to water, sanitation, agriculture and infrastructure, negatively impacting the health of the region's most marginalised.^33^ The constant influx of migrants across the region has challenged local government structures that are already struggling with large populations and inadequate social welfare infrastructure (employment, food distribution, housing and functioning safety networks), some of which are also exacerbated by climate-induced vulnerabilities.^9^ In addition to the public health and socioeconomic challenges the region faces, LAC is highly vulnerable to the effects of climate change and environmental degradation with severe developmental consequences. The region is facing the impact of a heating planet that has caused more frequent and prolonged droughts, extreme heat waves, increased precipitation, devastating floods, severe storms, wildfires, and landslides.^34^ Such events have caused the loss of housing, infrastructure, food crops, livestock farming, and communities' livelihoods. Disasters triggered 23.7 millions of internal displacements (IDs) globally in 2021,^2^ with 449,000 people displaced in Brazil, whilst hurricane Laura, Eta and Iota caused additional 2.7 million displacements across the region.^35^ According to the World Bank projections, climate change could force 216 million people to move within their own countries by 2050, 17.1 million only in Latin America.^36^

## Regional climate vulnerabilities and interconnections with migration and displacement

The *El Niño Costero* (coastal El Niño) phenomenon that affects Peru and Ecuador to a greater extent, is considered natural cyclical, but it could be exacerbated by climate change^37^ and cause increased migration. In Peru, approximately 300,000 people, including migrants, have been displaced by this phenomenon, yet the migrant population was not protected by national programs.^38^^,^^39^ Central America also faces a dramatic situation, as four of its seven countries ranked among the 20 most severely affected by extreme weather events.^40^ At the heart of Central America lies the Dry Corridor, spanning across 44% of the land area of Guatemala, Honduras, El Salvador and Nicaragua and is a prominent symbol of the sub-region climate vulnerability. Predominantly rural, the Dry Corridor area is home to approximately 11.5 million individuals whose livelihoods heavily depend on agriculture and subsistence farming. Prolonged droughts and volatile precipitation negatively affect crops, livestock, and exacerbate existing vulnerabilities rooted in poverty, violence, food insecurity and socioeconomic distress.^41^ By analysing data on crop productivity and migration trends between Mexico and the United States, research shows a significant correlation: as crop yields decrease due to climate change, more people tend to migrate from rural Mexico to the US. The study underscores the potential for climate-driven migration to become a major issue globally, affecting regions beyond Mexico.^42^ Emigration is often the last resort, despite all the uncertainty of what may come next.^43^ In 2021, there were nearly 600,000 asylum seekers and refugees from this region, mostly in Costa Rica, Mexico, and the United States.^44^ International migration from Guatemala, Honduras, and El Salvador to North America has been exacerbated by prolonged droughts, which have intensified the strain on food resources in these highly impoverished regions.^45^ The Caribbean region is also severely impacted by the effects of climate change (for the specific case of Haiti see box 1). The State of the Climate in Latin America and the Caribbean 2022 report reveals,^34^ among other impacts, that coastal areas of LAC and Small Island Developing States (SIDS) are experiencing a more rapid rise in sea levels compared to the global average. Current projections suggest that Haiti alone may face a grim scenario, with approximately 100 thousand individuals at risk due to a 1-m surge in sea levels by the end of this century.^8^^,^^46^ These events are already taking a toll on public health, causing economic repercussions, livelihood loss, and forcing people into migration and internal displacement. For instance, research reveals that Hurricane Maria caused a substantial impact, equivalent to 260% of Dominica's annual Gross Domestic Product (GDP), resulting in the displacement of as much as 27.3% of its population.^8^ Furthermore, exposure to extreme heat in Central America and the Caribbean is disproportionately affecting young, unskilled women. This has been linked to a twofold increase in the likelihood of migrating to provincial capitals. The root cause lies in temperature fluctuations affecting industries predominantly occupied by women, such as seam stressing, ultimately leading to income loss, thus amplifying vulnerabilities.^47^

**Box 1** **Haiti and the interrelatedness of social, structural, political, and ecological determinants of migrant's health.**

Haitians are the second-largest LAC migrant group living abroad after Venezuelans. From Chile to Mexico, people from Haiti are amongst the most disadvantaged migrant populations, facing hardship and discrimination, and the consequences of migration deterrence policies.^66^

Driven by violence, hunger, and scarce opportunities, Haitians are one of the top nationalities crossing the Darien Gap.^67^ Of those who made the journey, 105,000 were detained at the US–Mexico border^66^^,^^68^, ^69^, ^70^ while 36,000 of them were deported in early 2023.^71^ Despite Haiti's resistance and self-liberation from the colonial past, the present domestic and international forces make sure that Haiti's’ independence and development remains limited. The current and historical structural and institutional violence, fuelled by foreign interests, heavily shapes Haiti's social, political, and economic landscape. The latter, intensified by climate change and chronic environmental degradation, further exacerbates the situation within and across the LAC region. Examples include the notorious development-induced migration in Haiti resulting from the Péligre Hydroelectric and Irrigation System in the 1950's, both forcing thousands from their land, degrading the soil, and enriching those abroad. Centuries of land erosion and water systems degradation either from direct exploitation of fields by plantation is central to the current internal and cross-border migration as well as loss of livelihoods within Haiti.^72^^,^^73^

Haiti, with a population of 11.7 million, struggles to meet the population's need as severe inequality, poverty, and political instability, compounded by disasters—such as the 7.0 magnitude earthquake in January 2010 and Hurricane Matthew in 2016—exacerbate vulnerability and despair. Life expectancy is only 63 years, ten years below the global average, with high maternal and under-five mortality rates (732/100,000 and 59/1000 live births).^74^ Health expenditure is just 3.22% of GDP, reflecting the lack of investment in healthcare and infrastructure, with only 0.2 physicians and 0.4 nurses and midwives per 1000 people. Floods and storms account for most IDs in the country, with weak governance, and infrastructure,^75^ leaving 96% of the population at environmental risk.^75^ Between 2010 and 2022, over 36 natural events displaced more than 2.3 million people, triggering significant waves of distress migration across LAC.

The 2010 earthquake marked a significant displacement event, affecting more than three million people. This event killed over 222,570 people, and injured 300,000 more, with over 1.5 million IDs.^76^ The same year, homes, government and social structures were destroyed, possibly triggered by inadequate climate-resilient infrastructure and emergency preparedness plans. A subsequent Cholera outbreak, fuelled by heavy rains and landslides, resulted in an additional 10,000 Cholera-related deaths and 73,000 rain-related IDs, worsening Haiti's socioeconomic conditions.^77^^,^^78^

Haiti faces frequent climate displacement. Hurricane Matthew in 2016 displaced nearly 351,000 people, with 20% in need of humanitarian aid. This disaster strained Haiti's already fragile health and social systems, prompting another 105,000 individuals to seek jobs in countries such as Brazil and Chile.^78^^,^^79^ From 2020 to 2021 between 182,000 and 200,000 lived in Chile and Brazil, respectively, likely influenced by the 2021 earthquake which displaced around 220,000 people.^79^

Subsequent socio-political unrest in Haiti disrupted the energy supply, in turn impacting health delivery and humanitarian assistance amid Cholera outbreaks, the COVID-19 pandemic and gang-related violence. In 2022, The Internal Displacement Monitoring Centre reported that conflict and violence internally displaced 171,000, with an additional 15,000 displaced by climate events.^71^

The case of distress migration within and outside Haiti illustrates climate change's impact on the social, political, and economic landscapes. With rising temperatures, rainfall, earthquakes, and drought, across transit and host countries. Climate change may force more Haitians northwards, facing harsher living and working conditions.

## Climate change, migration and health

Understanding the relations between climate change, migration and health remains challenging^11^ and has been increasingly recognised as a priority in the global health research agenda.^48^^,^^49^ However, the interactions between these three phenomena, which are complex in themselves, are manifold. Migration can provide significant health benefits by enabling people to escape extreme weather conditions, access better healthcare services, find economic opportunities, reduce exposure to violence, and improve access to clean water and sanitation. These factors contribute to overall well-being and quality of life, especially in contexts of climate change and socio-economic pressures. Available research shows that the dynamics between anthropogenic climate change and migration may lead both to health benefits and to severe risks for individuals and populations,^50^, ^51^, ^52^, ^53^, ^54^, ^55^, ^56^ depending on the specific population, and context. Physical, geographic, and climatological aspects like heat, droughts, and precipitation play an essential role, just as ecological factors like the prevalence of vector-borne diseases. However, the health impact of extreme weather events and long-term environmental changes is essentially shaped by social, economic and political factors, with marginalised groups being the most at risk of suffering the negative health impacts caused by the climate crisis.

### Immediate climate-related impacts on migrant health

Extreme weather events affect individuals and populations according to their level of exposure and protection. All the social determinants of health risks associated with heat and all the other physical extremes that increasingly undermine the habitability of our planet, will at first and most severely affect marginalised people who live in precarious social, economic and health conditions, frequently facing severe discrimination to access health services, housing, food, education, among others, in precarious living conditions. This is often the case for distressed migrants who not only recently fled countries like Venezuela and Haiti, but also for the victims of internal displacement due to violence and conflict, or rural-to-urban migration in countries like Colombia, Peru, and Brazil decades ago. Historically, the inhabitants of informal settlements in high-risk areas for landslides like Lima, São Paulo or Medellín, with little access to clean water and sanitation, are to a large extent internally displaced.^57^ Moreover, migrants are often forced to make their living working in the so-called DDD-jobs (“dirty, dangerous, and demeaning”)^58^ for example in construction and agriculture, with high levels of informality and precarious occupational protection. Weak health systems and reduced access to health services in transit or destination countries increase the health burden and vulnerability of distressed migrants in the climate crisis.

### Climate change intensifies health risks for refugees, migrants, and displaced groups

**Fig. 1:**
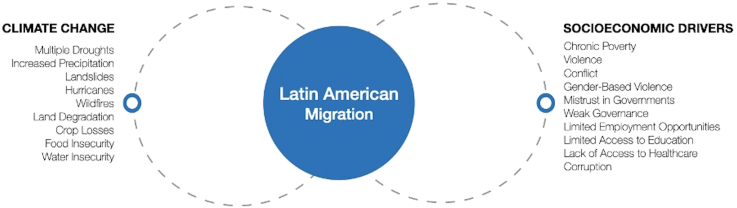
Interconnectedness of climate change and socioeconomic drivers for migration in Latin America.

Besides the immediate effects (e.g. the heat waves on individuals’ health) slow onset climate change and environmental degradation add an additional layer of adversity to pre-existing vulnerabilities. In a 2020 review examining human mobility, climate change and health, McMichael illustrated that climate-related events contribute to an increase in malnutrition, respiratory and cardiovascular issues, vector-borne, waterborne diseases and infectious diseases, as well as heightened prevalence of heat-related illness.^52^ The impact on mental health has also been shown to be severe.^59^^,^^60^ Climate change thus not only aggravates and accelerates migratory pressure in many regions and communities, it also aggravates and accelerates the deterioration of health determinants before, during and after the migration process. Consequently, migrants suffer the double burden of climate-induced health risk and of the continuously more restrictive migration policies in LAC (and globally): the detrimental health effects of prolonged transit, detention, violence, expulsions, and the precarious lives at the margins of societies, with often limited access to health care, are reinforced by the direct and indirect consequences of the climate crisis (See Fig. 1).

### Migration as final adaptation strategy in response to climate-induced health threats

Confronted with rising sea levels, hurricanes or severe droughts that turn formerly fertile lands into deserts like Central America's “Northern Triangle”, migration is often the sole survival option.^5^ However, whether emigration actually offers a viable solution with real perspectives for a healthy and safe future, or merely results in displacement without dignified resettlement, ultimately hinges on the prevailing social, economic and political context.

Populations affected by poverty and the often-intersecting dimensions of discrimination and social exclusion based on ethnicity, race, gender, and migration background are at the highest risk of becoming internal or international distress migrants. Among the multiple reasons, for example, that make people join the migrant caravans traversing Central America and Mexico to the United States, climate-related environmental pressures play an important role.^61^ The UN Special Rapporteur on extreme poverty and human rights already warned in 2019 of the emergence of a “climate apartheid scenario”, when the “wealthy pay to escape overheating, hunger and conflict, while the rest of the world is left to suffer”.^62^

Three studies from a project in Atlanta, USA, exemplify a robust approach to understanding the intersection of climate change, migration, and health advocating for comprehensive, community-centred approaches to adaptation to face climate challenges. Laney et al. explored how climate-related vulnerabilities impact Latin American migrants' health and migration decisions, capturing the nuanced experiences and social determinants influencing their adaptation.^63^ Similarly, Lane et al., investigated the structural and environmental stressors faced by these migrants post-resettlement, revealing disparities such as high rates of mould and food insecurity exacerbated by climate factors.^64^ On the other hand, Herrera et al. further contextualise these issues by identifying climate-related drivers of migration among Latin American and Caribbean immigrants, highlighting the neglected health burdens, and calling for improved healthcare access and surveillance to address these vulnerabilities effectively.^65^

## Migration, health, and climate justice

Individuals and families who flee from unbearable living conditions as a direct or indirect consequence of climate change and environmental degradation are affected in multiple terms: by losing their homes, by the increased vulnerability of climate and migration-related hazards as distress migrants, and the burden of a completely uncertain future for themselves and their loved ones.^80^

Considering the glaring imbalance between the responsibility for anthropogenic climate change and its impact, all efforts to mitigate and prevent climate-related suffering and destruction need to be based on the principle of climate justice—also in the field of health. In Latin America, climate justice has become a concept of integration and mobilisation,^81^ linking the environmental, social, and ethical dimensions of the sustainability, development and struggle of indigenous peoples and other groups disproportionately affected by climate change.^82^

LAC is one of the most unequal regions in the world, with stark income inequalities, wealth concentration, rural-urban disparities, unplanned urbanisation, and limited investments in infrastructure and human development.^83^ These features reflect the region's persisting colonial legacy that contributes to systemic racism, poor education, power imbalances, targeted rights violations, stigmatisation and social-environmental vulnerabilities.^30^, ^31^, ^32^^,^^84^ Throughout the region, extractive economies—e.g. in agriculture and mineral extraction—with little regard for ecological, social, or economic sustainability as well as the needs of local—often indigenous—populations have left a serious burden of environmental and social degradation, including violence and forced migration. Within the “globalised systems of wealth extraction and profiteering”, identified recently as one key feature of “contemporary colonialism”^85^ impacting global health, large parts of LAC are suffering the legacies of such activities. Embracing climate justice despite its challenges represents an unprecedented opportunity to promote sustainable development for all, considering the threats posed by the current economic and political paradigms.^86^

## Indigenous communities: at the crossroads of climate change, migration, and health

Approximately 50 million Indigenous people live in LAC and despite making up for eight percent of the region's total population, they account for 14% of the poor and 17% of the extremely poor^87^ and suffer from worse health outcomes when compared to non-Indigenous.^88^ Since the beginning of European colonisation, Indigenous communities have suffered the negative impacts of colonisation, territorial invasions, environmental degradation, land dispossession, conflict, diseases, and displacement.^89^ Paradoxically, Indigenous-managed lands have lower deforestation rates than other areas, underscoring the pivotal role these communities play in safeguarding the environment and its biodiversity.^90^ Despite being good stewards of the forests and their minimal contribution to climate change, Indigenous people are heavily burdened by its consequences and find themselves caught in a climate injustice conundrum.^91^

Notwithstanding the limited research and data about the climate-migration nexus and Indigenous peoples' health in LAC, recent research about deforestation and climate change in the Brazilian Amazon indicate that the concurrent impacts of Amazon savannisation and climate change are notably linked to extreme risks affecting human health and overall well-being in potentially irreversible ways.^92^ Indigenous peoples’ strong connection and reliance on natural resources are disproportionately threatened by the impacts of environmental degradation caused by mining activities, oil extraction, land grabbing and dispossession illegal logging, deforestation, and violence.^93^, ^94^, ^95^, ^96^ Predominantly, activities such as mining, deforestation, and poaching affecting Indigenous lands and communities are conducted by non-Indigenous rather than by Indigenous peoples themselves. The frequency and scale of these unlawful actions are escalating at a distressing pace, posing serious threats to their health, well-being, and survival.

Recent data from Brazil show that illegal mining operations on Indigenous territories and other legally protected areas have hit a record high in the past few years. Between 1985 and 2020, the mining areas in the country grew sixfold, mostly located in the Amazon region. Moreover, from 2010 to 2020, the area occupied by mining activities within indigenous lands increased by 495%, coinciding with the encroachment upon their territories. Such pattern underscores the historical and ongoing exploitation and violation of Indigenous rights, exacerbating their vulnerability and adding another dimension influencing their decision to migrate.^90^^,^^97^, ^98^, ^99^ The Yanomami Indigenous in Brazil are facing a humanitarian crisis driven by such unprecedented levels of deforestation and illegal mining (including exposure to harmful high levels of mercury used for it), logging and land grabbing that has caused livelihood losses, food insecurity, malnutrition, epidemics of malaria, diarrhoea and mercury poisoning, threatening their health and future survival.^97^^,^^98^

As the climate crisis causes ecosystem distress, threatening natural resources that are essential to Indigenous people's identity, livelihoods, food sources and cultural practices, Indigenous peoples face increasing pressure that includes lifestyle changes, including forced migration from their traditional territories as an adaptation strategy. Indigenous people's identity and well-being rely on the collective access to their ancestral territories and cultural practices, the latter being considered a determinant of health for this population.^100^^,^^101^ Available research underscores the mental health impacts of environmental degradation and forced migration on Indigenous communities. The enduring trauma of traditional land losses and the erosion of cultural practices have been linked to increased rates of mental distress and substance abuse.^89^^,^^102^^,^^103^

The consequence of lifestyle changes coupled with mental distress also contribute to growing rates of obesity, and diabetes, and other diseases.^87^ Elderly Indigenous, considered guardians of their heritage, face increased health risks as they either refuse to leave their lands or are physically unable to withhold the migration journey, remaining trapped in deforested areas with limited access to natural resources, food, water and diseases brought by illegal miners, land grabbers and others threatening their survival and livelihoods.^104^ Most Indigenous people who migrate to the cities in search of a better life, continue to face marginalisation and significant barriers to accessing healthcare, education, housing, and social benefits.^100^ However, some native peoples of South America have successfully adapted to the effects of climate change by merging ancestral knowledge with climate-resilient initiatives.^105^^,^^106^ Climate change and environmental degradation have a disproportionate impact on Indigenous women in LAC, as drought and rainfall impact the agricultural sector—the most important employment sector for women in this region—affecting their ability and agency to care for their families and communities, particularly children.^107^ In Chile, the impacts of climate change on natural resources are significant obstacles for Mapuche and Peheunche Indigenous women in charge of agricultural activities to feed their communities.^108^ Consequently, these Indigenous women are forced to migrate to seek other modes of subsistence, increasing their vulnerability and risks to their health.^108^ Moreover, the climate crisis has contributed to growing levels of gender-based violence, compounded by cultural loss, food insecurity, land loss, further exacerbating existing economic, health and social inequalities and vulnerabilities. Gender-based violence experienced by Indigenous women occurs at various levels, manifesting within their households and communities, and extends into interactions at the community and state level, involving both state and non-state actors. This persistent violence is deeply rooted gender inequalities and unequal power structures, racism, and marginalization enabled by the legacy of colonialism and alarming levels of impunity by perpetrators. Existing evidence underscores that the manifestation of violence against Indigenous girls and women cannot be separated from broader contexts of discrimination and exclusion experienced by Indigenous peoples.^109^^,^^110^

## Recommendations

The severe health impact of the dynamics between climate change and migration has important implications in LAC and beyond. With all the advancements in regional policy arenas to address climate change, migration, and health separately,^8^^,^^28^^,^^111^^,^^112^ it is of paramount importance to develop an integrative, transdisciplinary approach and to bridge the gap between evidence and action (See Table 1 for the recommendations summary).

**Table 1:** Summary of strategic recommendations for addressing climate-driven migration and health impacts in Latin America.

Policy needs	Policy recommendations	Recommended strategies
Legal & Policy Frameworks	• Establishment of legal national and regional frameworks that recognise climate-induced migrants and ensure their protection, assistance, and relocation through a rights-based approach.	• Development of laws and policies that address issues such as forced displacement, resettlement, and access to basic services in destination countries.
Funding & Finance	• Secure appropriate budget from governmental institutions and increased private funding to support adaptation measures and efforts to address the root causes of climate change at local level.	• Foster public-private partnerships to diversify economic-employment opportunities for communities that are heavily dependent on agricultural practices. • Support international collaboration to boost funding for conservation efforts and sustainable development.
Capacity Building	• Collaboration between various sectors (academia, NGOs, governments, and civil society organizations) to co-design train and develop programs to strengthen the capacity of indigenous and other vulnerable communities to better adapt to the effects of a changing climate.	• Active engagement of local actors to co-create tailored training strategies and projects to increase the capacity of affected communities.
Data & Research	• Strengthen dialogue between academia, local organisations and policymakers to design research to generate evidence on the nexus between climate change and migration. • Development of multi-stakeholder initiatives to share data and information in real-time, respecting individuals’ privacy and migrants’ rights	• Collaboration for the development of cross-border public health records through the creation of a health-specific database to support the needs of migrants.
Policy Frameworks for Indigenous Peoples	• Reinforcement, protection and guarantee of Indigenous land protection legislation to safeguard Indigenous rights. • Development of adaptation strategies targeted to address the disproportionate impacts of climate change on Indigenous communities and other vulnerable groups. • Design and strengthen socioeconomic protection programs, targeted to indigenous communities. Governments should develop and implement social protection programs to support Indigenous communities affected by climate change.	• Set up a strong legal group that protects and increases the vigilance over the Indigenous land protection legislation. • Highlight the significance of developing strategies with communities to support affected populations in diversifying their livelihoods. This includes leveraging innovative tools, such as technology-driven solutions, to enhance livelihood optimization, improve access to safe water, and mobilize resources for constructing climate-resilient infrastructure. • Programs should include cash transfers, food assistance, improved healthcare access and other forms of support that respond to co-define needs.

Countries need to incorporate heath, migration and mobility into their climate strategies and policies, and vice versa, with a full commitment^113^ to the highest attainable standard of health of all people—migrants and non-migrants, nationals, and foreigners—rooted in climate justice principles. This includes a revision of migration deterrence policies that keep migrants in situations of prolonged vulnerability for suffering the direct and indirect health impacts of extreme weather events and other climate-induced risks.

### Climate adaptation

Multilateral efforts have been made towards climate adaptation, mitigation, resilience-building strategies, disaster preparedness and response. Such efforts need to be strengthened by a systematic incorporation of the migration and health dimensions, with the establishment of a rights-based legal framework, increased funding, and stronger coordination among international, national, and local stakeholders. Involuntary migration as an adaptation strategy to climate change needs to be prevented.

Measures should be taken to address the root causes of climate change to reduce the necessity for involuntary migration, ensuring that individuals are not forced to relocate due to climate-related hazards. This involves strengthening local resilience, providing adequate resources and support to at-risk communities, and enhancing adaptive capacity.

### Participation and empowerment

The active engagement of affected individuals and communities in the design and implementation of policies and solutions need to be ensured. Power and resources need to be shifted to empower migrants^114^, ^115^, ^116^ and displaced persons by implementing targeted policies that provide them with access to education, job training, and legal support. Additionally, integrating their knowledge and experiences into community planning and decision-making processes can enhance their agency. For example, governments and NGOs can collaborate to create mentorship programs and support networks. Specific initiatives could include setting up local advisory councils composed of displaced persons to guide policy development and implementation, ensuring that their unique insights and needs are addressed directly.

The LAC region has a strong tradition in social mobilisation initiatives that have contributed to more inclusive policies and broader citizens’ participation.

The CityAdapt project is an initiative executed by the United Nations Environment Programme (UNEP) and funded by the Global Environment Facility that pioneered the implementation of Ecosystem-based Adaptation (EbA) strategies in urban adaptation planning across LAC. The core objective of CityAdapt is to enhance urban climate resilience through the integration of natural ecosystems into urban planning and development. The initiative intends to promote biodiversity and enhance the standard of living for urban dwellers while simultaneously mitigating the negative effects of climate change, such as heatwaves, flooding, and water scarcity, by utilizing the natural advantages of ecosystems. The CityAdapt initiative places a strong emphasis on stakeholder interaction at all levels and involves the active involvement of national, regional, and local government bodies, promoting a cooperative and inclusive strategy for climate resilience. The project has demonstrated that multi-stakeholder and participatory techniques are necessary for the successful implementation of EbA plans, ensuring that the demands and viewpoints of many stakeholders are taken into account. The project's positive impact has been achieved in cities in Mexico, Jamaica, and El Salvador. The project emphasised the importance of stakeholder engagement across multiple levels, from local communities to regional and national authorities, fostering a collaborative approach to climate resilience. Valuable lessons gained from this project and other initiatives underscore the potential of participatory and multi-stakeholder approaches in enhancing climate resilience, while offering valuable insights for future adaptation initiatives.^117^

### Equity

Indigenous populations and other historically marginalised populations need to be placed at the centre of initiatives that address climate, migration, and health as they bear the highest brunt of the unequal effects of climate change. Indigenous peoples have developed a synergistic relationship with the surrounding environment, and the inclusion of Indigenous perspectives into Western approaches can enhance equity in policies and contribute to knowledge generation and enhancement. Moreover, climate justice cannot be achieved without gender equality. This requires the development of gender-sensitive policies, the empowerment of women leaders through training and mentorship, gender-responsive budgeting, support for women's organisations working on climate justice, and investment in research to understand gendered impacts and design equitable interventions. These actions will ensure that the rights and contributions of both Indigenous populations and women are acknowledged and integrated into efforts to achieve a sustainable and just future.

### Research and data

The last few years have seen a significant increase in the number of research studies on climate change, migration, and health.^118^ However, it is still imperative to address this broad topic in a more systematic way, adopting a transdisciplinary approach that focus simultaneously on the drivers, determinants and solutions. Studies that apply participatory approaches, amplifying their voices and agency to affected individuals and communities, are urgently needed.^119^ Another significant challenge is the scarcity of collaborative data collection and real-time information sharing. It is of paramount importance that more data are gathered, using strict privacy and rights-based approaches, to enable for a more accurate understanding and mapping of the relationship between climate change, human displacement, and health in LAC.

## Conclusions

Incorporating climate justice for distress migrants into health research and policy is imperative for addressing the health impacts of climate change and ensuring a just transition towards a sustainable future for all in LAC. However, research and policies on climate change and health often overlook the needs of migrant populations, who, for multiple reasons, are at heightened risk of experiencing the health impacts associated with both the drivers of climate change (e.g. extractivist economies) and the direct and indirect health risks. A comprehensive approach thus needs to address the root causes of environmental degradation and displacement, including access to health, and safeguarding the dignity and rights of affected populations.

Overall, addressing climate-induced migration in LAC requires strong multi-sectoral collaborations, including public and private sectors, civil society organisations, and academic institutions. Governments must urgently develop tailored adaptation strategies, implement (or reinforce) land-use regulations, ensure the protection of indigenous territories, and strengthen socioeconomic protection programs, especially for the most marginalised. Academic institutions can contribute and offer key support in research and implementation, and also provide technical assistance and co-create training programs with affected communities.

In this Personal View, we provide a comprehensive analysis of the complex interplay of climate change–health–migration in LAC. The complexity of these issues, the significant regional diversity (both within and between countries) and their influences on such issues mean that not all aspects and nuances were covered.

Moreover, our manuscript is predominantly qualitative, built upon available literature, including reports, peer-reviewed publications, and authors’ experiences. Nonetheless, we have been able to identify, describe and analyse key trends, challenges and opportunities which can contribute to enhanced knowledge and generate questions that can support future research. We also summarised a set of recommendations that can guide and support practices in these topics.

Despite the challenges posed by climate change and displacement for migrants’ health and well-being, there is hope for a sustainable future. Our political systems have the ability to promote socioeconomic benefits and foster prosperity and human development for both nations of origin and destination if they can manage migration in a rights-based and sustainable manner while adhering to international standards, goals, and regulations. It is up to new policies/new political views/new political leaders to act and enable a better future for all.

## Contributors

CB–bibliographic review, original draft, writing, editing.

MK–bibliographic review, writing, editing. ACS–bibliographic review, writing, editing. SF–bibliographic review, writing, editing.

DW–bibliographic review, writing, editing.

DB–bibliographic review, writing, editing.

KR—bibliographic review, writing, editing.

JCA–bibliographic review, writing, editing.

MYG–bibliographic review, writing, editing.

## Declaration of interests

We, the authors, declare no competing interests.

## Appendix A Supplementary data

Translated abstract
