# Supplementary material for: Climate change, migration, and health: perspectives from Latin America and the Caribbean
Source: Lancet Reg Health Am. 2024 Oct 23;40:100926. doi: 10.1016/j.lana.2024.100926 (PMC11703577; doi:10.1016/j.lana.2024.100926)
Supplement: Translated abstract [file mmc1.docx]

**Editor note:** *This translation in Spanish and Portuguese was submitted by the authors and we reproduce it as supplied. It has not been peer reviewed. Our editorial processes have only been applied to the original abstract in English, which should serve as reference for this manuscript.*

**Spanish and Portuguese Translated Abstracts**

**Resumen**

Este artículo explora la compleja relación entre el cambio climático, los patrones migratorios y los impactos en la salud en América Latina y el Caribe (ALC). Aunque el grave impacto del cambio climático en la salud en ALC es ampliamente reconocido, el artículo resalta los múltiples efectos, a menudo negligenciados, sobre la migración y el bienestar de los migrantes. En particular, estos impactos abarcan la pobreza, la inseguridad alimentaria y de agua, así como resultados adversos en la salud física y mental. Nuestro trabajo, guiado por un marco basado en los derechos humanos, busca identificar tendencias clave, desafíos y oportunidades para contribuir a un mayor conocimiento y formular preguntas que impulsen investigaciones futuras. Al enfatizar la necesidad de esfuerzos colaborativos entre sectores, incluyendo entidades públicas y privadas, la sociedad civil y las instituciones académicas, pretendemos abordar las intersecciones del cambio climático, la migración y los impactos en la salud en la región. Este enfoque prioriza las necesidades de los más vulnerables, incluidos los migrantes, estableciendo un marco para la mitigación y adaptación que garantice resultados equitativos.

**Resumo**

Este artigo explora a complexa relação entre as mudanças climáticas, os padrões migratórios e os seus impactos na América Latina e Caribe. Embora o impacto severo das mudanças climáticas na saúde nesses países seja amplamente reconhecido, o artigo lança luz sobre os múltiplos efeitos frequentemente negligenciados na migração e no bem-estar dos migrantes.

Esses impactos abrangem pobreza, insegurança alimentar e hídrica, e resultados adversos para a saúde física e mental. Nosso artigo, guiado por uma abordagem baseada em direitos, tem como objetivo identificar as principais tendências, desafios e oportunidades que possam contribuir para o aumento do conhecimento e gerar questões que apoiem pesquisas futuras. Ao enfatizar a necessidade de esforços colaborativos entre setores, incluindo entidades públicas e privadas, a sociedade civil e instituições acadêmicas, buscamos estabelecer as bases para enfrentar as interseções e nuances entre mudanças climáticas, migração e impactos na saúde na região. Essa abordagem prioriza as necessidades dos grupos mais vulneráveis, incluindo os migrantes, estabelecendo um marco para mitigação e adaptação que assegure resultados equitativos.
